# Monitoring of granite quarries using deep learning and UAV photogrammetry in Bengaluru, India

**DOI:** 10.1371/journal.pone.0334493

**Published:** 2025-11-06

**Authors:** Oussama Himmy, Thanh Thi Nguyen, Prem Jose Vazhacharickal, Andreas Buerkert

**Affiliations:** 1 Organic Plant Production and Agroecosystems Research in the Tropics and Subtropics, Universität Kassel, Witzenhausen, Germany; 2 Global Development Department, College of Agriculture and Life Sciences, Cornell University,; 3 Rural-Urban Center, University of Agricultural Sciences GKVK, Bengaluru, India; USACE ERDC: US Army Engineer Research and Development Center, UNITED STATES OF AMERICA

## Abstract

Granite quarrying, a cornerstone of the construction industry in South India, yields significant economic benefits but poses substantial environmental and social challenges, including land degradation, dust pollution, alternation of the water regime, and harsh working conditions. Rapid urban expansion has escalated granite demand in many countries, intensifying quarrying activities. This trend is particularly pronounced in Bengaluru, India, where rural-urban transformation causes concerns about environmental sustainability and social-ecological consequences of urban resource mining. This study proposes an innovative multi-modal framework to monitor granite quarrying in Bengaluru by combining deep learning with a 2024 dry-season multi-date Sentinel-2 composite for quarry segmentation and UAV SfM-MVS photogrammetry for volumetrics. We benchmark five CNN architectures—U-Net, PSPNet, DeepLabV3 + , FCN, and EMANet. In-area development results peaked with DeepLabV3+ (F1 ≈ 94.6%, IoU ≈ 89.7%), while an external, geographically independent audit established PSPNet as the most robust model (F1 = 93.4% [95% CI 90.8–95.9], IoU = 87.6%) with significantly fewer errors than alternatives (McNemar tests, FDR-adjusted p < 0.001). Applying the best model across the region yielded 252 candidates; 227 quarries were confirmed via field checks and sub-meter imagery, spanning 740 hectares. UAV photogrammetry at the Prasannacharipalya site (0.046 m grid; LoD95 masking), yielded a combined lowering volume of 9 280 051 m³ (acceptance area 97.2%; 95% CI ± 17 864 m³, 0.19%). The satellite-to-UAV pipeline enabled automated, scalable quarry footprint mapping with site-level volumetric quantification, offering actionable evidence for environmental management and oversight of quarrying in the quickly-urbanizing study region.

## 1. Introduction

Granite quarrying is the process of extracting granite, an igneous rock, from the earth for construction, decoration, and sculpture [1,2]. It is prominent among natural stones globally, with a high demand in domestic and international markets. However, extraction comes with substantial environmental and social impacts [3–5].

Quarrying activities significantly alter landscapes. They involve the removal of soil and bedrock by drilling, cutting, and blasting techniques that break rocks into manageable pieces [6,7]. This entails the loss of topsoil, can result in a barren and unstable land surface [8–10], may cause soil erosion, and ultimately leads to the formation of pits and quarries that may pose safety risks and disrupt water drainage patterns [11]. In Odeda Local Government Area of Ogun State, Nigeria, quarrying activities have led to a decrease in areas covered by light forest, from 637.282 hectares in 1984 to 326.517 hectares by 2014, indicating significant land cover changes due to quarrying [12]. The extraction and processing of materials also generates dust, noise, and vibrations, which may adversely affect the environment and human health. Dust can cover vegetation thereby reducing photosynthesis, and affect air quality for nearby communities [12,13]. On the positive side, in South India many old, abandoned quarries traditionally serve as tanks, providing water for animals and irrigation during the dry season [14].

Remote sensing has emerged as a powerful tool for monitoring various mining activities, offering a cost-effective, efficient, and comprehensive approach to assessing the environmental impacts of mining operations [15,16]. The availability of publicly available datasets such as Landsat and Sentinel, along with the advancement of analysis-ready data archives [17], has facilitated widespread access to remotely sensed information. This, in turn, enables researchers and policymakers to quantify human-induced disturbances caused by mining activities [18].

Delineating mining areas is a critical task within the broader scope of Land Use/Land Cover (LULC) mapping, which is pivotal for environmental monitoring, planning, and management. Traditionally, pixel-based classification has dominated this process, yet it often lacks spatial context and yields “salt-and-pepper” noise and boundary fragmentation [19,20]. This is not only an aesthetic issue: fragmented rims inflate false positives/negatives near quarry edges, bias areal estimates, and propagate error to any downstream change/volume indicators built from those polygons. Object-based image analysis (GEOBIA) partially mitigates these errors via segmentation prior to classification, but the-crafted features and thresholds can generalize poorly across heterogeneous quarry landscapes triggering modern end-to-end, context-aware deep learning approaches [21,22].

In recent years, Convolutional Neural Networks (CNNs) have become standard for extracting mining footprints from multispectral imagery. Despite initial challenges -such as limited pre-labeled datasets and the difficulties in analyzing multispectral and hyperspectral imagery -representative studies report competitive performance across mining task and sensors. For artisanal/small-scale mining (ASM), a Sentinel-2 multispectral CNN detected sites reliably at a regional scale, outperforming traditional baselines and demonstrating policy relevance for unregistered sites [23]. For open-pit delineation on high-resolution imagery, an EMANet + CRF workflow achieved PA ≈ 98.1% and mIoU ≈ 89.5%, while another dense-encoder variant reported precision ≈ 97.7% and IoU ≈ 72.1%, illustrating both the potential and the variability of results across datasets and designs [24]. For tailings-related segmentation, optimized DeepLabv3 + variants have achieved mIoU ≈ 63% under challenging low-contrast conditions, underscoring the value of multi-scale context and boundary-aware decoders yet also the remaining generalization challenges [25]. These design choices address, in measurable terms, the shortcomings of pixel-based methods: multi-scale context reduces spurious speckle over bright soils and construction sites; skip connections and decoders stabilize quarry rims -the features that govern area/perimeter -and attention modules suppress noise in heterogeneous benches/haul roads. This is why recent mining-specific studies have shifted toward CNNs for footprint mapping.

While satellite-scale segmentation maps may show where quarries are and how they expand, regulators and planners also need to know how much material has been extracted. In this context, Unmanned Aerial Vehicles (UAVs) have emerged as effective means for mine mapping and feature quantification, producing centimeter-scale Digital Surface Model (DSM)/ orthophoto products suitable for cut-and-fill analysis. UAV SfM–MVS has matured into a low-cost, high-resolution topographic tool with well-documented accuracy characteristics; best-practice guidance highlights how careful flight geometry, inclusion of oblique imagery, and sensible ground control points (GCP) or RTK/PPK geotagging mitigate systematic errors and support robust change detection and volumetrics. Multiple studies in quarries and stockpiles have shown that UAV-derived volumes compare favorably with GNSS/terrestrial surveys, validating UAVs as a quantitative complement to satellite detection. In terms of precision, UAV SfM–MVS typically achieves absolute mapping accuracies of ~1–3 × GSD when supported by well-distributed GCPs or RTK/PPK image geotags (i.e., a few centimeters for 2–5 cm GSD), whereas no-GCP configurations can exhibit absolute biases at decimeter- to meter-scale [26–28]. Moreover, recent frameworks explicitly connect satellite triggers to targeted UAS acquisition for rapid updates of Digital Elevation Models (DEM), reinforcing the synergy between regional DL mapping and local 3D measurement [29–32]. This study addresses the above limitations with a multi-modal, end-to-end framework to monitoring granite quarrying activities around the South Indian megacity of Bengaluru. We integrated deep learning techniques with satellite imagery and UAV photogrammetry to: (i) develop a robust deep learning framework for automated quarry detection from multispectral satellite data withcontext-aware CNNs to stabilize quarry boundaries and reduce speckle, (ii) benchmark U-Net, PSPNet, FCN, DeepLabv3 + , and EMANet as strong complementary baselines for quarry segmentation. Finally, (iii) we integrate UAV photogrammetry to independently quantify excavation volume (cut-fill) and to cross-check segmentation geometry at centimeter GSD- explicitly reporting uncertainty and discussing practical constraints. This integrated design allows for actionable, scalable monitoring while retaining the precision required for on-site environmental management.

## 2. Materials and methods

### 2.1. Study area

**Fig 1:**
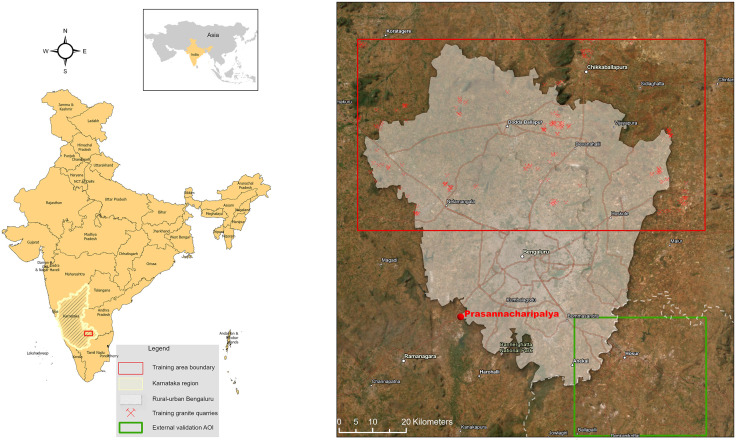
Study area in rural-urban Bengaluru, India, and training/ validation data boundary. Map was created using ArcGIS (version: 3.5) from ESRI (http://www.arcgis.com/) under the institutional ESRI site license of the University of Kassel. Basemap satellite image accessed from World Imagery ESRTI Tile Layer. Credits: Esri, Earthstar Geographics, TomTom, Garmin, FAO, NOAA, USGS, © OpenStreetMap contributors, and the GIS User Community.

According to official data, India, a globally significant granite producer, boasts 3.314 mining leases covering 38 major minerals on a total formal lease area of approximately 306.398 hectares [33]. Hereby, the states of Karnataka, Andhra Pradesh, Tamil Nadu, and Rajasthan contribute about 75% of the country’s total granite production. Bengaluru, the capital of Karnataka, has been chosen as a case study area due to the rapid expansion of granite quarrying and its major apparent impact on the local environment, vegetation, and people’s livelihoods (Fig 1). Known as the India’s ‘Silicon Valley’ and ‘City of Lakes’ Bengaluru’s burgeoning urban development and construction needs have accelerated the demand for granite. This increased demand has resulted in substantial land-use changes often at the expense of natural vegetation and habitats [4].

**Fig 2:**
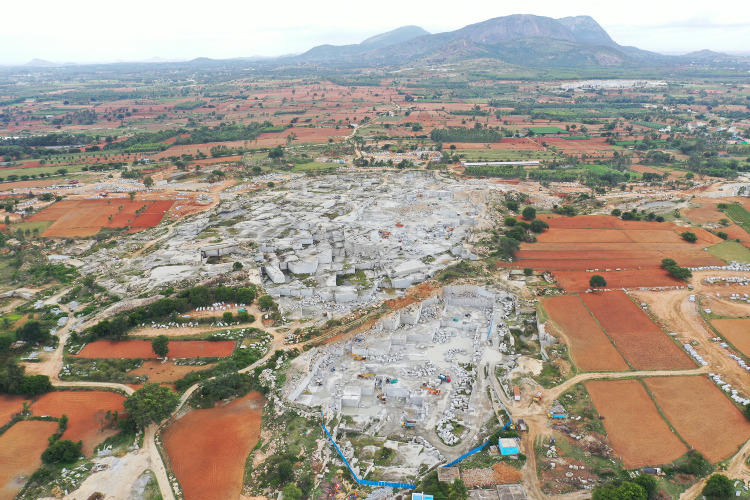
Aerial picture of Koira granite quarry in Bengaluru, India, taken by the authors in July 2023.

Bengaluru’s granite (Fig 2) results from the ancient Precambrian rocks foundation of the Peninsular Gneissic Complex (PGC). This complex includes granites, gneisses, and magmatites formed billions of years ago [34]. Over time, weathering has created many soil types ranging from red laterite to clayey deposits.

The environmental effects of granite quarrying in Bengaluru are pronounced. Excavation processes lead to extensive deforestation and the clearance of native vegetation. Soil erosion, water pollution from quarrying activities, and dust emissions challenge air and water quality in the region [35,36]. Further, granite quarrying practices disrupt the hydrological balance and jeopardize nearby water bodies while providing at the same time the possibility to establish new tanks for irrigation or leisure [37–39]. Often, however, the adverse impacts of these quarries extend beyond their immediate vicinity, jeopardizing both urban and rural areas [40].

This study’s focus area encompasses districts around Bengaluru with known active granite quarries or clear evidence of ongoing or historical quarrying. This approach ensured comprehensive coverage of relevant quarry sites and aligns with available baseline data that can support subsequent analysis (Fig 1).

### 2.2. Data acquisition and preprocessing

#### 2.2.1. Elevation data.

Elevation data were obtained from the NASADEM Digital Elevation Model (DEM), a 30-meter resolution produced by a full reprocessing of Shuttle Radar Topography Mission (SRTM C-band InSAR; Feb-2000) data with improved phrase unwrapping and vertical control using ICESat. Remaining voids are primarily filled with refined ASTER GDEM v3. Distributed in WGS84 horizontal and EGM96 (vertical) datum and covers ~60° N to 56° S in June 2024. This product was accessed via Google Earth Engine (GEE) https://earthengine.google.com/. Published evaluations report typical absolute vertical errors of ~6–10 m Root Mean Square Error (RMSE) for NASADEM, with accuracy varying by terrain ruggedness, slope/aspect, vegetation, and region; consequently, precision is not uniform globally [41,42]. C-band penetration into vegetation can bias elevations in forested areas; users commonly observe reduced biases after reprocessing, but residual effects can persist locally [43,44].

The DEM was resampled using bilinear interpolation to match the spatial resolution of other datasets, ensuring consistent dimensionality across all inputs. This elevation layer helped in refining quarry detection and volume estimation processes by capturing surface morphology and improving the accuracy of subsequent analyses.

#### 2.2.2. Sentinel-2 satellite data.

ESA’s Sentinel-2 MSI provides high-resolution multispectral imagery for accurately detecting granite quarries. Its spatial resolution (10–20 m) and broad spectral range (visible, near-infrared, and short-wave infrared bands: Table 1), enable clear discrimination between quarry surfaces and surrounding landscapes. Granite’s distinctive spectral response, particularly in the visible, Near-Infra Red (NIR), and Short-Wave Infrared (SWIR) range, aids in automated quarry detection using deep-learning models [45].

**Table 1:** Characteristics of used spectral bands of Sentinel-2 images for the study of granite mines around Bengaluru, South India.

Bands	Resolution (m)	Wavelength (nm)	Description
**B1 - Aerosols**	60	443.9	Not used
**B2 – Blue**	10	496.6	Used
**B3 – Green**	10	560	Used
**B4 – Red**	10	664.5	Used
**B5 – Red edge 1**	20	703.9	Used
**B6 – Red edge 2**	20	740.2	Used
**B7 – Red edge 3**	20	782.5	Used
**B8 – NIR**	10	835.1	Used
**B8A – Red edge 4**	20	864.8	Used
**B9 – Water vapor**	60	945	Not used
**B11 – SWIR 1**	20	1613.7	Used
**B12 – SWIR 2**	20	2202.4	Used

For this study, Sentinel-2 L2A images from the dry season of 2024 were accessed via Google Earth Engine (GEE). We deliberately limited data selection to the dry season (December–March) to maximize clear-sky observations in this monsoonal climate and to reduce vegetation-related spectral confusion. Dry winter months in India have substantially lower cloud fractions than monsoon months, and dry-season selections are widely adopted for LULC/mining mapping. To ensure data quality, images with cloud cover exceeding 20% were excluded, and the s2cloudless algorithm [46] was applied to remove remaining clouds and shadows. A median composite of all valid observations was generated, minimizing noise and highlighting stable quarry features. As aerosols (Band 1) and water vapor (Band 9) bands contributed minimally to land surface characterization, they were excluded. Six 20 m bands were resampled to 10 m resolution using bicubic interpolation [47]. To enhance detection accuracy, topographic information from the NASADEM was integrated with the Sentinel-2 composite, enabling the model to leverage both spectral and terrain-related features [48,49].

**Fig 3:**
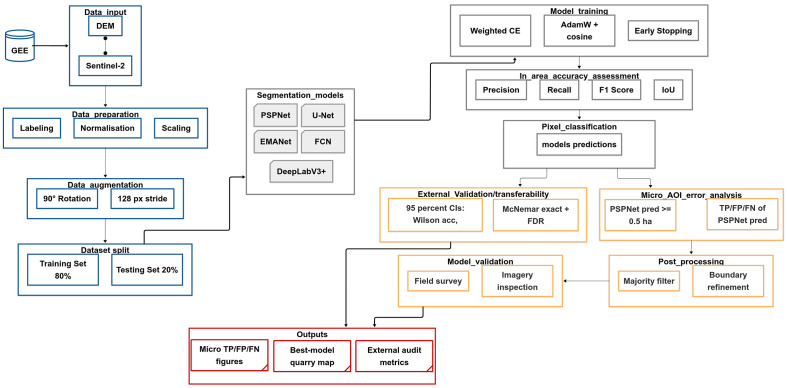
Mining site detection workflow based on deep learning for the study of granite mines around Bengaluru, South India.

For our study, we selected an area of approximately 93 km by 52 km north of Bengaluru because of its abundance of granite quarries (Fig 1). Quarry polygons used for model training and validation were manually digitized in ArcGIS Pro (v3.3) by a single annotator with expertise in mining operation and practical experience in interpreting high resolution imagery of mining environment. Digitization relied primarily on high-resolution imagery from Google Earth Pro supplemented by Maxar satellite imagery [50]. Each delineated quarry underwent a secondary review using multi-temporal imagery (Sentinel 2) to confirm extraction activity and distinguish between active and abandoned sites. Ambiguous cases were cross-referenced by field surveys. The (Fig 3) composite was tiled into 256 x 256 pixel patches (10 m GSD; 2.56 × 2.56 km per patch) with a 128-pixel stride. To improve rotational invariance, we applied 90° rotations [51]. The dataset (1384 patches) was split into an 80−20 ratio for training and testing, respectively, ensuring proper model evaluation on unseen data while maintaining sufficient training samples for optimal learning [52,53]. This systematic approach to data preparation established a solid foundation for developing a generalizable model capable of accurate quarry detection across diverse landscapes.

#### 2.2.3. Drone imagery.

High-resolution drone imagery was acquired on 8 March 2024 at the Prasannacharipalya granite-quarry site under clear skies (air temperature ≈ 25°C; relative humidity ≈ 24%; wind speed < 3 m s ⁻ ¹), conditions chosen to minimize motion blur and illumination change. A DJI Mavic 2 Pro drone equipped with a 20-megapixel Hasselblad L1D-20c camera flew a nadir grid flight generated in Dronelink [54], at an altitude of 200 meters above ground level (agl), with 75% forward/ 70% side overlap to ensure comprehensive site coverage and accurate photogrammetric processing [55–57]. In total 343 images (5472 × 3648 px) were acquired during the 35-min survey. Georeferencing relied on the drone’s onboard dual-frequency GNSS (absolute positional accuracy ≈ 1–2 m).

The high-quality imagery obtained from this mission served as the primary input for generating a detailed 3D model of the study site. Subsequent processing of these images using Pix4Dmapper software v.4.9 [58] yielded a high-resolution orthomosaic and a precise 3D model. To ensure compatibility between the pre-quarry and post-quarry data, we resampled the DEM using bilinear interpolation to match the resolution of the DSM generated from our 3D model. This technique is particularly effective in maintaining the integrity of the elevation data while allowing for a more refined representation of the terrain [59].

#### 2.2.4. Deep learning-based quarry detection.

We leveraged state-of-the-art deep learning-based semantic segmentation architectures to identify mining sites from satellite imagery. Five primary models, U-Net,PSPNet, DeepLabV3 + , FCN, and EMANet were employed and benchmarked, following the workflow below (Fig 3).

The selection of U-Net, PSPNet, DeepLabV3 + , FCN, and EMANet was based on their complementary architectural strengths which have translated into state-of-the art quarry-segmentation results across multiple recent benchmarks, outperforming earlier CNNs and matching heavier transformer hybrids [60].

U-Net’s encoder-decoder structure preserves fine spatial details by combining high-resolution features from early network layers with deeper contextual representations [61]. The model’s proven capability is to achieve high performance with limited training data, as evidenced by the mean Intersection over Union (mIoU) score of 83.5%. This design makes it particularly well-suited for delineating the detailed boundaries characteristic of quarry sites. PSPNet incorporates pyramid pooling to capture multi-scale global context [62], facilitating more robust detection of mining patterns spread over larger areas. DeepLabV3+ [63,64] leverages Atrous Spatial Pyramid Pooling and a lightweight decoder to capture global context without sacrificing edge sharpness, achieving the highest F1/IoU once larger multisensory datasets are available [65].

FCN [66], canonical fully convolutional baseline allowed for early end-to-end dense prediction (useful reference for gains due to modern context/attention blocks).

EMANet replaces full self-attention with an expectation-maximisation block cutting memory by an order of magnitude [67]; when paired with a CRF it delineated open-pit mines at 89.48% MioU 15 k × 15 k scenes [24]. These covered the main families (skip-decoder, multi-scale pooling, dilated (atrous) convolutions/ ASPP, attention, and baseline). Heavier transformer hybrids were not included to keep computing cost and inference latency compatible with the workflow.

All networks used a ResNet34 backbone (pre-trained on ImageNet) for a strong initialization for feature extraction, enhancing efficiency and mitigating gradient vanishing issues through residual connections.

Inverse frequency weighting was integrated into the cross-entropy loss function to address the class imbalance, emphasizing the accurate detection of less frequent quarry pixels [68]. This approach prioritized the precise prediction of minority mining sites. We used the AdamW optimizer [69] with weight decay to reduce overfitting. Hyperparameters were tuned through iterative experimentation, using an initial learning rate of $1\times 10^{-4}$ and a cosine annealing schedule. Batch sizes of 4–8 were chosen to balance computational demands and model performance, and early stopping based on validation loss ensured resource-efficient training [70]. Training and inference were conducted on an NVIDIA RTX 4070 GPU supported by a 13th Gen Intel® Core™ i7-13620H CPU. Models were trained on the 80% training set and evaluated on the non-overlapping 20% test set. We computed per-pixel precision, recall, F1 score (Identical to Dice coefficient), and Intersection over Union (IoU) and aggregated them over test patches – reflecting performance from multiple perspectives [71, Table 2].

**Table 2:** Segmentation Accuracy Metrics (*TP = True Positive; FP = False Positive; FN = False Negative*) for the study of granite mines around Bengaluru, South India.

Metric	Equation
**Precision**	$\frac{TP}{TP+FP}\backslash )$
**Recall**	$\frac{TP}{TP+FN}\backslash )$
**F1 Score**	$2\;\times \;\frac{Precision\;\times Recall}{Precision+Recall}\backslash )$
**Intersection over Union (IoU)**	$\frac{TP}{TP+FP-FN}\backslash )$

For proportions, we applied Wilson 95% confidence intervals; for F1 we computed bootstrap 95% Cis (2 000 resamples of TP/FP/FN) [72]. Pairwise model differences at the same points were tested using exact two-sided McNemar on discordant errors with Benjamini-Hochberg FDR across all pairs [73,74].

To quantify generalization beyond the training area, we defined an external AOI (green area of interest in Fig 1) in South-eastern Bengaluru. We formed two strata (quarry/non-quarry) and sampled 200 points per stratum (n = 400). Each point was visually interpreted on sub-meter imagery (quarry = 1; other = 0). All five models were sampled at the same points without interpolation, allowing for paired tests. This follows recommended practice for design-based accuracy assessment with probability samples [75].

For spatially explicit error analysis, we chose a micro-AOI (~1–3 km²). Quarries were polygonised and filtered to ≥ 0.5 ha, then rasterized (snap/cell size of the training grid) to a binary ground-truth mask. For each model, we computed TP/FP/FN rasters and pixel-wise confusion counts. To further improve segmentation results, morphological operations such as opening and closing were applied to remove noise and fill gaps, refining the quarry boundaries detected.

Validation was carried out through (1) direct field-based observations and measurements at selected sites and (2) comparisons with high-resolution Google Earth imagery to verify areas inaccessible for field validation.

#### 2.2.5. Estimation of extracted granite volume.

The volume of extracted materials at the Prasannacharipalya mining site was determined using a photogrammetric workflow based on our high-resolution drone imagery. Following a preliminary quality check to discard blurred or poorly exposed images, Pix4D Mapper software was employed to process the data. The workflow began with a Structure from Motion (SfM) procedure [29], which identified and matched tie points across overlapping images and performed a bundle adjustment to refine camera orientations. Subsequently, a Multi-View Stereo (MVS) algorithm converted the sparse point cloud into a dense point cloud with an average density of 250 points per square meter [76,77]. From this dense point cloud, a high-resolution Digital Surface Model (DSM) was generated with a ground sampling distance of 5 cm per pixel.

For volume change detection, the UAV-derived DSM was differenced against a reference Digital Elevation Model (DEM) that represented the pre-extraction topography. This reference DEM was reconstructed from historical topographic data and undisturbed terrain near the mining area. Prior to differencing, we co-registered the UAV-DSM to the reference DEM over a mask of stable bedrock to remove residual horizontal/vertical biases and minor tilt using the Nuth & Kääb method [78], thereby minimizing systematic offsets and providing the residuals used to quantify the co-registration variance.

To examine scale effects on the volume estimate, we conducted the analysis at multiple grid sizes. The reference DEM was first resampled to match the working resolution of the DSM, and both the DSM and the reference DEM were then coarsened to a common 1 and 5-meter resolution, and a cut-and-fill analysis was executed in Python environment to compute volumetric differences.

For error propagation, we followed DEM-of-Difference (DoD) best practice if z_1_ and z_2_ are the elevations in the DSM and (resampled) DEM and σ_z1_, σ_z2_ their standard deviations (assumed to be independent), the per-cell elevation-difference uncertainty is:


$$\sigma_{\Delta z\;=\;\sqrt{\sigma_{z_{1}}^{2}+\;\sigma_{z_{2}}^{2}+\;\sigma_{coreg}^{2}}}\mathrm{\backslash ]}$$ (1)

We applied a 95% level-of-detection threshold ($L_{O}D_{\text{9}\text{5}}=\text{1}.\text{9}\text{6}\;\sigma_{\Delta z}$) to exclude changes below detection. Volumetric uncertainties were then propagated by (i) summing independent per-cell variances and (ii) a correlated formulation that accounts for spatial error structure via an empirical correlation length, following geodetic/DoD practice [79,80]. To avoid over-confidence on steep facets, LoD95 was spatially varied across slope bins (0–5–10–15–25–35–90°) by increasing σ_z2_ with slope; DoD cells were accepted only where |Δz| ≥ LoD95 and within the quarry masks.

As an additional robustness check, we performed a Monte-Carlo (MC) experiment on a 1 m grid in which the post-coregistration DSM was repeatedly perturbed by sub-pixel horizontal (dx, dy) and vertical (dz) jitters drawn from the measured coreg residuals. DoD and volume were recomputed per draw to produce sampling distributions and 95% MC confidence intervals [81].

## 3. Results and discussion

### 3.1. Detection and Segmentation Performance

Across the tested architectures, internal (in-distribution) performance was uniformly high (Table 3) in the training area of northern Bengaluru. DeepLabV3+ achieved the strongest balance (F1 ≈ 94.6%, IoU ≈ 89.7%), with PSPNet (F1 ≈ 94.2%, IoU ≈ 89.1%) and U-Net (F1 ≈ 93.6%, IoU ≈ 88.0%) close behind. EMANet and FCN trailed modestly but remained credible baselines. This ranking is consistent with the architectural biases of the models: multi-scale context aggregation (PSPNet’s pyramid pooling; DeepLab’s ASPP) tends to improve region coherence and suppress local texture confusion, while skip-decoder designs (U-Net) emphasize edge fidelity and small-object recovery. These observations mirror the original reports: PSPNet’s pyramid pooling explicitly aggregates global cues at multiple scales, DeepLabv3+ increases receptive field via atrous convolutions without sacrificing borders, and U-Net’s skip connections recover fine details from early layers [62,64]. Because the split is not geographically independent, we treat Table 3 as a development benchmark (descriptive). Still, the near-ceiling values confirm that modern CNNs can reliably map quarries at 10 m when trained and validated in-region, consistent with reports that context modules (pyramid/atrous) improve segmentation over early baselines [82].

**Table 3:** In-area performance of five deep learning architectures for granite quarry segmentation in Bengaluru, South India.

Model	Precision (%)	Recall (%)	F1 score (%)	IOU (%)
**U-Net**	93.3	94.0	93.6	88.0
**PSPNet**	95.7	92.8	94.2	89.1
**DeepLabV3+**	94.02	95.10	94.55	89.7
**EMANet**	91.08	92.31	91.69	84.6
**FCN**	93.07	92.77	92.92	86.8

On the geographically independent audit, models diverged (Table 4). PSPNet retained high performance with tight uncertainty: F1 = 93.4% (95% CI 90.8–95.9), IoU = 87.62% (TP = 177, FP = 23, FN = 2, TN = 198). The other models were more conservative—higher precision, lower recall—and therefore achieved lower F1/IoU (FCN F1 = 74.49%, IoU = 59.56%; DeepLabV3+ 61.97%/45.05%; EMANet 54.23%/37.36%; U-Net 49.40%/32.97%). Paired McNemar tests on discordant errors confirmed that PSPNet yielded significantly fewer errors than every alternative (FDR-adjusted p < 0.001), establishing a statistically supported rank ordering. This behavior matches broader evidence that explicit context modules (pyramid/atrous) are more robust when background heterogeneity increases (urban fringe, laterite, bare soil) and aligns with recent mining-RS studies using Sentinel-2 CNNs for open-pit/ASM detection [23,83,84]. Operationally, employing a recall-prioritizing model like PSPNet is preferable when the cost of missing active/illegal sites is high; the modest FP surplus is manageable in analyst triage. Where false alarms are costly, a two-stage workflow (contextual veto with DEM/indices) can keep precision high [85,86].

**Table 4:** External audit (n = 400): Accuracy ± 95% Wilson CI, Precision, Recall, F1 ± 95% bootstrap CI, and IoU.

Model	Accuracy (95% CI)	Precision (%)	Recall (%)	F1 (95% CI)	IoU (%)
**PSPNet**	93.8 [90.9–95.7]	88.50	98.89	93.4 [90.8–95.9]	**87.62**
**FCN**	81.5 [77.4–85.0]	96.46	60.89	74.5 [68.8–79.6]	59.56
**DeepLabV3+**	75.0 [70.5–79.0]	96.47	45.81	62.0 [55.0–69.1]	45.05
**EMANet**	71.5 [66.9–75.7]	95.77	37.99	54.2 [46.4–61.6]	37.36
**U-Net**	69.5 [64.8–73.8]	95.24	33.52	49.4 [41.0–57.3]	32.97

**Fig 4:**
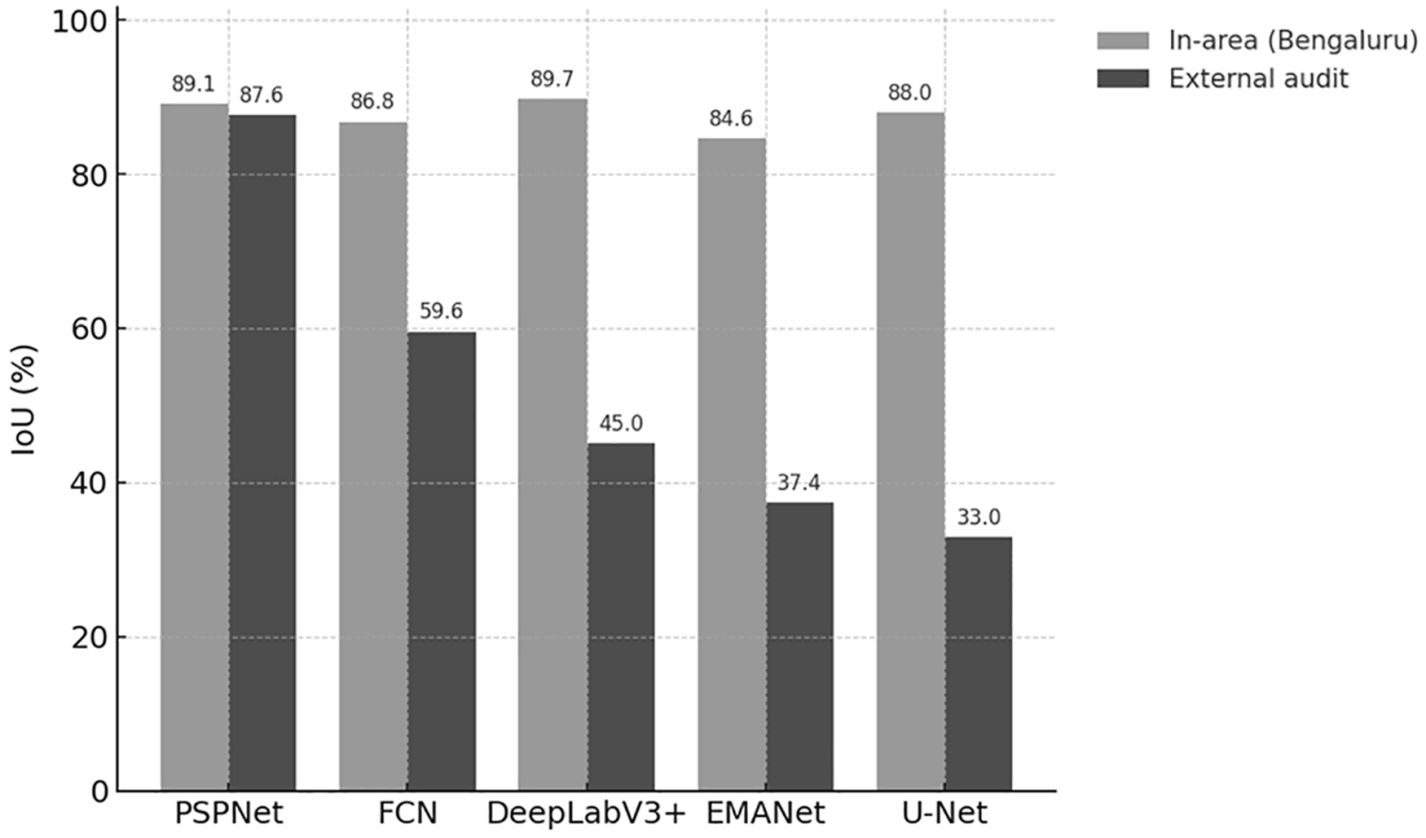
Generalization gap: bar pairs per model (in-area IoU vs external IoU).

Fig 4 summarizes the “generalization gap” visually: per model, bars show in-area IoU (Table 3) versus external IoU (Table 4). The drop is moderate for PSPNet, but more pronounced for DeepLabV3 + , FCN, EMANet, and U-Net – consistent with the notion that explicit context modules confer robustness beyond the training footprint.

**Fig 5:**
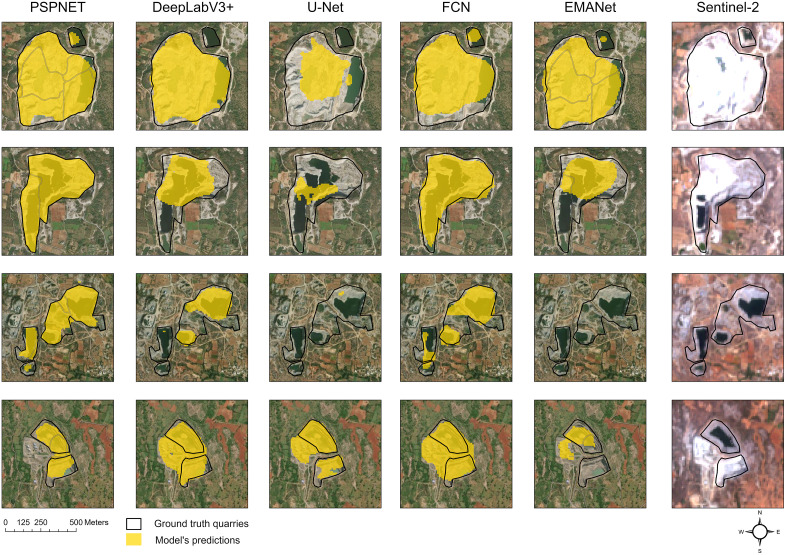
Qualitative performance in the *external* validation AOI (unseen during training) of PSPNet, DeepLabV3+ , U-Net, FCN, and EMANet of four granite quarries around Bengaluru, South India. Map was created using ArcGIS (version: 3.5) under the institutional ESRI site license of the University of Kassel, Germany (http://www.arcgis.com/). Basemap satellite image accessed from World Imagery ESRTI Tile Layer. Credits: Esri, Maxar, Earthstar Geographics, TomTom, Garmin, FAO, NOAA, USGS, © OpenStreetMap contributors, and the GIS User Community.

Consistent with the audit statistics in Table 4, PSPNet retains high recall and delineates irregular pits, whereas U-Net, DeepLabV3+, EMANet, and FCN are more conservative and miss small or occluded quarries (Fig 5).

**Fig 6:**
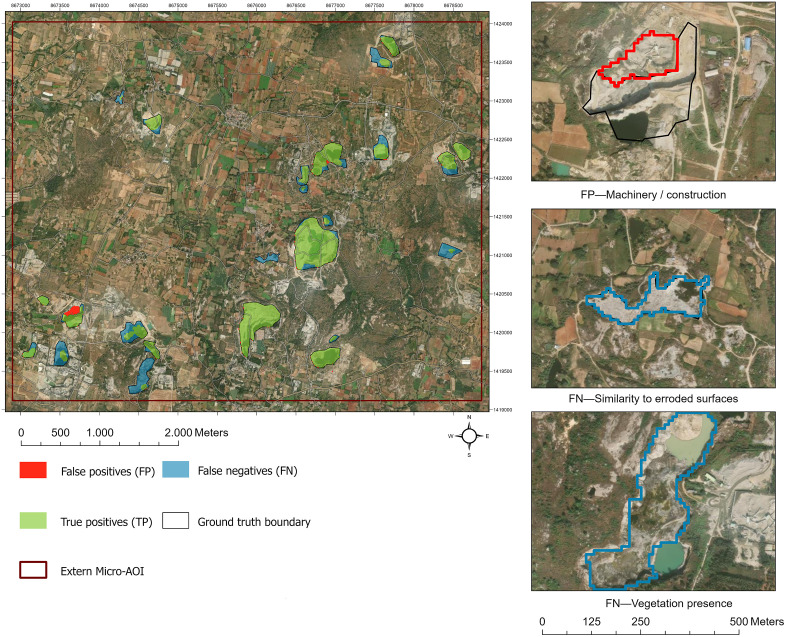
Micro-AOI TP/FP/FN overlays + the 3-panel failure cases for PSPNet model. Map was created using ArcGIS (version: 3.5) under the institutional ESRI site license of the University of Kassel, Germany (http://www.arcgis.com/). Basemap satellite image accessed from World Imagery ESRTI Tile Layer. Credits: Esri, Maxar, Earthstar Geographics, TomTom, Garmin, FAO, NOAA, USGS, © OpenStreetMap contributors, and the GIS User Community.

Pixel-wise confusion maps over a hand-edited micro-AOI reveal three interpretable, repeatable error modes (Fig 6): false positives over bright construction/storage yards and lateritic exposures (spectral/texture look-alikes to benches and spoil piles in VNIR/SWIR), false negatives for small (< 0.5 ha), vegetated, or water-filled pits (occlusion and size effects). Boundary raggedness along terraced faces (sub-pixel mixing at 10 m).

In the micro-AOI, PSPNet produced the most compact TP cores with fewer FN halos; FCN was thinner and more fragmentary; U-Net occasionally over-segmented rock outcrops at quarry margins. The three-panel failure in Fig 6 illustrates typical cases we observed.

**Fig 7:**
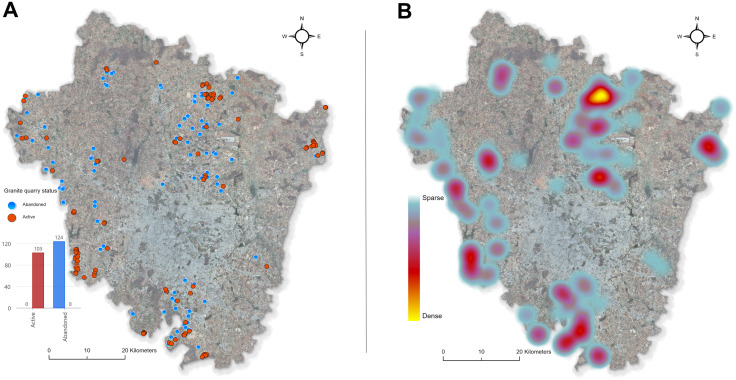
(A) Distribution of active and abandoned granite quarry in Bengaluru (South India) of 2024. **(B)** **Heatmap of granite quarry density.** It was created using ArcGIS (version: 3.5) under the institutional ESRI site license of the University of Kassel, Germany.

Applying the best model (PSPNet) across the rural and urban administrative boundaries of Bengaluru yielded 252 granite quarries 227 of which were confirmed via field checks and high-resolution imagery, covering an estimated 740 hectares (Fig 7). This validation resulted in a confirmation rate of 90%, reinforcing the model’s reliability in identifying quarries in previously unobserved regions. Among confirmed sites, 124 quarries were classified as abandoned based on indicators such as water accumulation, vegetation regrowth, and lack of recent excavation – an approach similar to post-classification refinement strategies used in mining land-use studies [23,87]. Conversely, 103 quarries displayed ongoing mining activities, evidenced by exposed rock, visible machinery, and detectable site alterations in sequential imagery, paralleling multi-temporal validation practices employed in mining footprint detection [24].

### 3.2. 3D model and volumetric analysis

**Fig 8:**
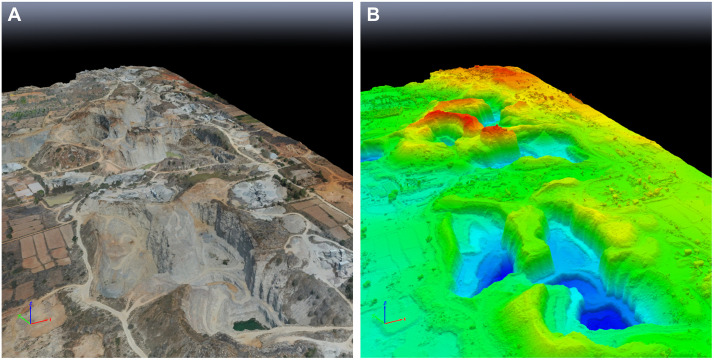
High-resolution 3D mesh of the Prasannacharipalya quarry near Bengaluru (South India), generated with Pix4Dmapper version 4.9.0 shows excavation areas and granite formations (A) Textured shader (B) Altitude shader (Red, Green, Blue). To this end authors’ drone images taken in July 2023 were mosaicked.

The 3D model of the granite quarry site (Fig 8) effectively captured the intricate terrain over an area of approximately 1.43 km^2^ with a Ground Sampling Distance (GSD) of 4.6 cm. The project successfully calibrated and geolocated all 343 images, achieving a mean reprojection error of just 0.166 pixels, underscoring the model’s precision. The resulting dense point cloud consisted of over 55 million points, with an average density of 30.4 points per cubic meter, facilitating the creation of a detailed digital surface model (DSM) and orthomosaic. Recent advances in geometry-aware point-cloud learning demonstrate centimeter-scale localization accuracy in similarly cluttered scenes [88]. This underscores the suitability of high-density point clouds for downstream quantitative tasks such as our cut-and-fill volume estimation.

Despite the absence of GCPs, georeferencing was accurate reflecting optimized internal camera parameters [89].

**Fig 9:**
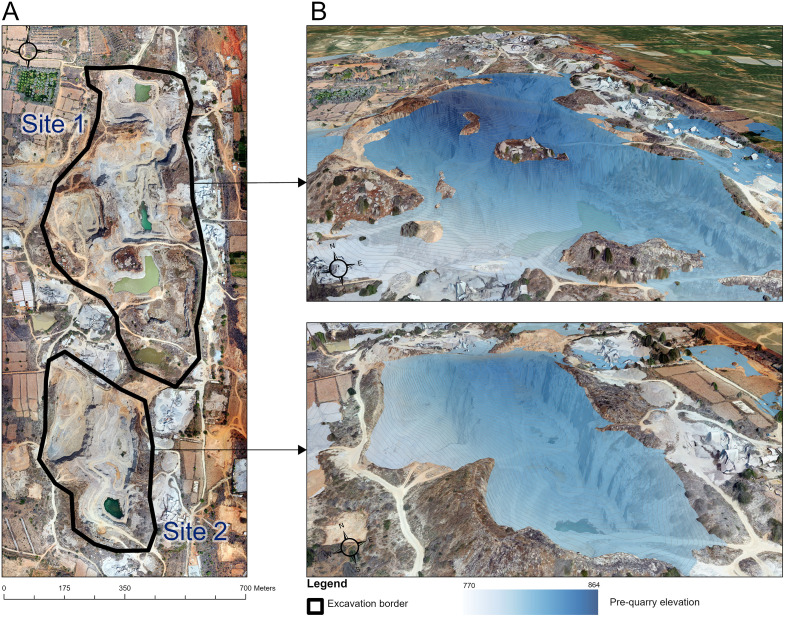
(A) High resolution mosaic of authors’ drone images taken in July 2023 showing the border of volumetric analysis of the Prasannacharipalya granite quarry near Bengaluru, South India and (B) textured 3D mesh showcasing the pre-quarry and post-quarry elevation.

The volumetric analysis of two extraction areas (Fig 9) within the granite quarry yielded quantitative insights into material removal and displacement after coregistration and parametric, slope-dependent LoD95, the DoD acceptance mask covered 96.8–98.1% of the mapped area across sites and resolutions. At the native UAV grid (0.046 m) the lowering components and their correlated 95% confidence intervals (CI) are presented in Table 4.

Repeating the full workflow at 1 m and 5 m yields lowering totals within +0.23–0.38% of the 0.046 m baseline (Table R1). Specifically, the combined lowering is 9 315 638 m³ at 1 m (+35 587 m³, + 0.38%) and 9 301 761 m³ at 5 m (+21 710 m³, + 0.23%). Correlated 95% CIs remain small (0.20–0.38% if lower) indicating that resampling contributes negligibly to the overall uncertainty compared with vertical accuracy and residual coregistration variance.

A MC jitter test at 1 m (sub-pixel dx/dy and dz drawn from the measured coreg residuals) yielded following a 95% CI: Site 1: ± 50 422 m³; Site 2: ± 27 722 m³; Combined. ± 77 970 m³ (≈ 0.84% of the MC mean lowering; Table 5). This independently corroborates the LoD-propagated CIs and shows that residual coreg errors have a minor impact; Table 6.

**Table 5:** Volumes and uncertainties associated with surface lowering at extraction sites of granite mines around Bengaluru, South India.

Grid (m)	Site	Lower (m³)	Raise (m³)	Acc.area (%)	95% CI (corr) on Lower (m³)	95% CI (% of Lower)
0.046	Site 1	6 025 558	−814 214	96.8	±14 534	0.24
0.046	Site 2	3 254 486	−350 482	98.1	±10 386	0.32
0.046	Combined	9 280 051	−1 164 696	97.2	±17 864	0.19
1.000	Site 1	6 046 914	−836 568	97.1	±15 539	0.26
1.000	Site 2	3 268 724	−365 667	98.1	±10 969	0.34
1.000	Combined	9 315 638	−1 202 235	97.4	±19 020	0.20
5.000	Site 1	6 037 016	−869 933	96.6	±17 246	0.29
5.000	Site 2	3 264 745	−394 254	97.9	±12 403	0.38
5.000	Combined	9 301 761	−1 264 187	97.0	±21 243	0.23

**Table 6:** Results of the Monte-Carlo experiment (lowering-only) at 1 m grid.

Site	Mean lower (m³)	σ (m³)	95% CI (m³)
**Site 1**	6 030 696	25 726	±50 422
**Site 1**	3 258 964	14 144	±27 722
**Combined**	9 289 660	39 781	±77 970

Based on an assumed average rock density of 2.7 g cm^-3^ the mass of the extracted granite amounts to 25.1 million metric tons (Mt); the 95% CI on mass is ± 0.048 Mt (scales directly with the 0.19% lowering CI). Laboratory and regional gravity data show that granites of the Peninsular Gneissic Complex span 2.60–2.75 g cm-^-3^ (mean 2.65 g cm^-3^) depending on feldspar–quartz ratio and degree of alteration [90]. Applying these end members to the extracted volume yields a mass range of 24.13–25.52 Mt, i.e., - 3.7% to +1.8% around the 2.70 g cm^-3^ estimate. This density-driven uncertainty has been included in Table 5 and the overall error budget. Future surveys combining drill-core density measurement and high-resolution gravity data could further constrain this parameter.

Our UAV-derived volume estimate represents a significant improvement in precision compared to data derived from traditional survey methods. Interestingly, the volume distribution was not uniform across the extraction area, with notably higher concentrations in the northeastern quadrant. This non-uniformity likely stems from the site’s unique geological features and the strategic extraction patterns employed by the quarry operators. The 3D model also revealed subtle variations in the quarry floor topography, contributing to a more accurate volume calculation by accounting for undulations that might have been overlooked in conventional 2D surveys.

### 3.3. Limitations and future research

PSPNet, despite its sophisticated pyramid pooling architecture, exhibits notable limitations in precisely delineating quarry boundaries, particularly in complex scenarios [91] involving abandoned mining sites. While the network excels at capturing multi-scale contextual information through its pyramid structure, it apparently struggles with detailed boundary information and smooth edges in challenging landscapes. This limitation became particularly evident at abandoned quarry sites where water accumulation interrupted the typical spectral signature of granite exposures.

For future work, it may be beneficial to expand the dataset used for training and validation hereby including a broader range of geographical areas and mining site characteristics. This could involve incorporating data from different regions or countries to improve the model’s adaptability. Also, transfer learning techniques could help leverage existing models trained on similar tasks to enhance performance in new contexts [92].

Another avenue for improvement is the integration of temporal analysis using time-series satellite imagery to monitor changes in quarry sites over time. This approach could provide valuable insights into mining activities and their environmental impacts. However, it might face significant practical challenges. The historical limitations of satellite data, with Landsat imagery only available from the 1990s onwards, coupled with a substantial gap in Google Earth coverage for Bengaluru between 1985 and 2011, create significant obstacles for comprehensive temporal analysis. This is particularly problematic as many regional quarries predate such temporal datasets, and the absence of consistent historical imagery makes it difficult to verify quarry evolution patterns.

The volume estimation of the granite quarry using advanced 3D modeling techniques faces several limitations that merit consideration. The absence of ground control points (GCPs) introduces potential uncertainties in absolute georeferencing accuracy, particularly for large-scale projects. Although a dense network of GCPs remains the gold standard for absolute georeferencing, in unregulated or artisanal quarry settings the installation, and repeated surveying of targets can be impractical or even unsafe due to unstable slopes, active blasting, and access restrictions. To reduce the absolute georeferencing error inherent in our GCP-free workflow, future campaigns will employ RTK-PPK-enabled UAVs, which adding it with a minimal GCP network improves vertical accuracy from decimeter to just ±2–4 mm and overall 3-D RMSE to <5 mm [93].

Moreover, the use of DEM data, with its relatively coarse resolution (30 m), potentially misses subtle topographic features crucial for accurate volume calculations in complex quarry terrains. Quarry change detection will also move from single-epoch snapshots to quarterly UAV campaigns, enabling sub-decimeter SfM point-cloud differencing and early warning of slope instabilities.

The lack of historical extraction records and reference data hinders the validation of the model’s accuracy. This limitation reflects a common challenge in studies involving industrial sites with complex histories or changes in operational management [94].

## 4. Conclusions

Our study demonstrates the effectiveness of integrating deep learning techniques with multi-temporal satellite imagery analysis and UAV photogrammetry to monitor granite quarrying activities. The PSPNet-based deep learning model achieved high accuracy in detecting and segmenting granite quarries, providing a reliable tool for large-scale monitoring of mining activities. Identifying 227 granite quarries covering 740 hectares highlights the extensive nature of quarrying in the surroundings of the southern Indian megacity of Bengaluru region and its potential environmental impacts.

The high-precision volume estimation using UAV photogrammetry offers valuable insights into the scale of granite extraction, with implications for resource management and environmental assessment. This integrated approach provides a robust framework for monitoring quarrying activities, offering decision-makers and environmental managers a powerful tool for sustainable land use planning and policy formulation.

Future research should focus on expanding the geographical scope of such studies, incorporating temporal analysis to monitor changes over time, and exploring the integration of additional data sources to enhance the accuracy and applicability of the model. This comprehensive approach to quarry monitoring has the potential to significantly contribute to sustainable mining practices and environmental conservation efforts in Bengaluru and beyond.
